# Characterization of the complete mitochondrial genome and phylogenetic analysis of *Coelophora saucia* (Mulsant, 1850) (Coleoptera: Coccinellidae)

**DOI:** 10.1080/23802359.2025.2571724

**Published:** 2025-11-10

**Authors:** Jing-Qiang Zhou, Yi Li, Xue-Fei Cai, Feng Li, Guo-Hua Huang

**Affiliations:** ^a^ College of Plant Protection, Hunan Provincial Key Laboratory for Biology and Control of Plant Diseases and Insect Pests, Hunan Agricultural University, Changsha, China; ^b^ Amway (China) Botanical R&D Center, Wuxi, China; ^c^ Amway (Shanghai) Innovation & Science Co., Ltd, Shanghai, China

**Keywords:** *Coelophora saucia*, mitogenome, phylogeny

## Abstract

*Coelophora saucia* is an important natural enemy insect in agricultural production. We determined the complete mitochondrial genome of the *C. saucia* by high-throughput sequencing, and the mitogenome was 18,068 bp in length, with a GC content of 20.9%, encodes 2 ribosomal RNA genes, 22 transporter RNA genes, 13 protein-coding genes and with a non-coding control region. A phylogenetic tree was constructed using the maximum likelihood (ML) method, and the results indicated that the *C. saucia* was most closely related to *Propylea japonica* and *Propylea quattuordecimpunctata.*

## Introduction

*Coelophora saucia* (Mulsant, 1850) belongs to the family Coccinellidae in Coleoptera. Its body is nearly round and black, with a white circular spot on each side of the pronotum and an orange-red elongated patch in the center of the forewings (Doeurk et al. 2024). *C. saucia*, mainly preying on aphids, whiteflies and other pests, is an important predatory natural enemy insect in agriculture, widely distributed in China (Dai 1990a). It is highly predatory, with a maximum daily predation of 256 individuals of aphids (Chen et al. 2024a), and plays an important role in agro-ecological regulation. *C. saucia* has 5–6 generations per year in the Jianghuai Region, overwintering and over-summering as an adult, with overlapping generations often occurring due to the long egg-laying period (Dai 1990b). Despite extensive studies on its life history, reproductive characteristics, and predation potential (Chen et al. 2024b), there remains a need for further characterization of its mitochondrial genome and phylogenetic relationships within the broader context of Coccinellidae.

In this study, we determined the complete mitochondrial genome of *C. saucia* and clarified its phylogenetic status by constructing a phylogenetic tree using the maximum likelihood method. The results will not only contribute to our understanding of the genetic diversity and evolutionary relationships within the Coccinellidae family, but also provide valuable information for pest management and agroecological conservation.

## Materials

**Figure 1.**
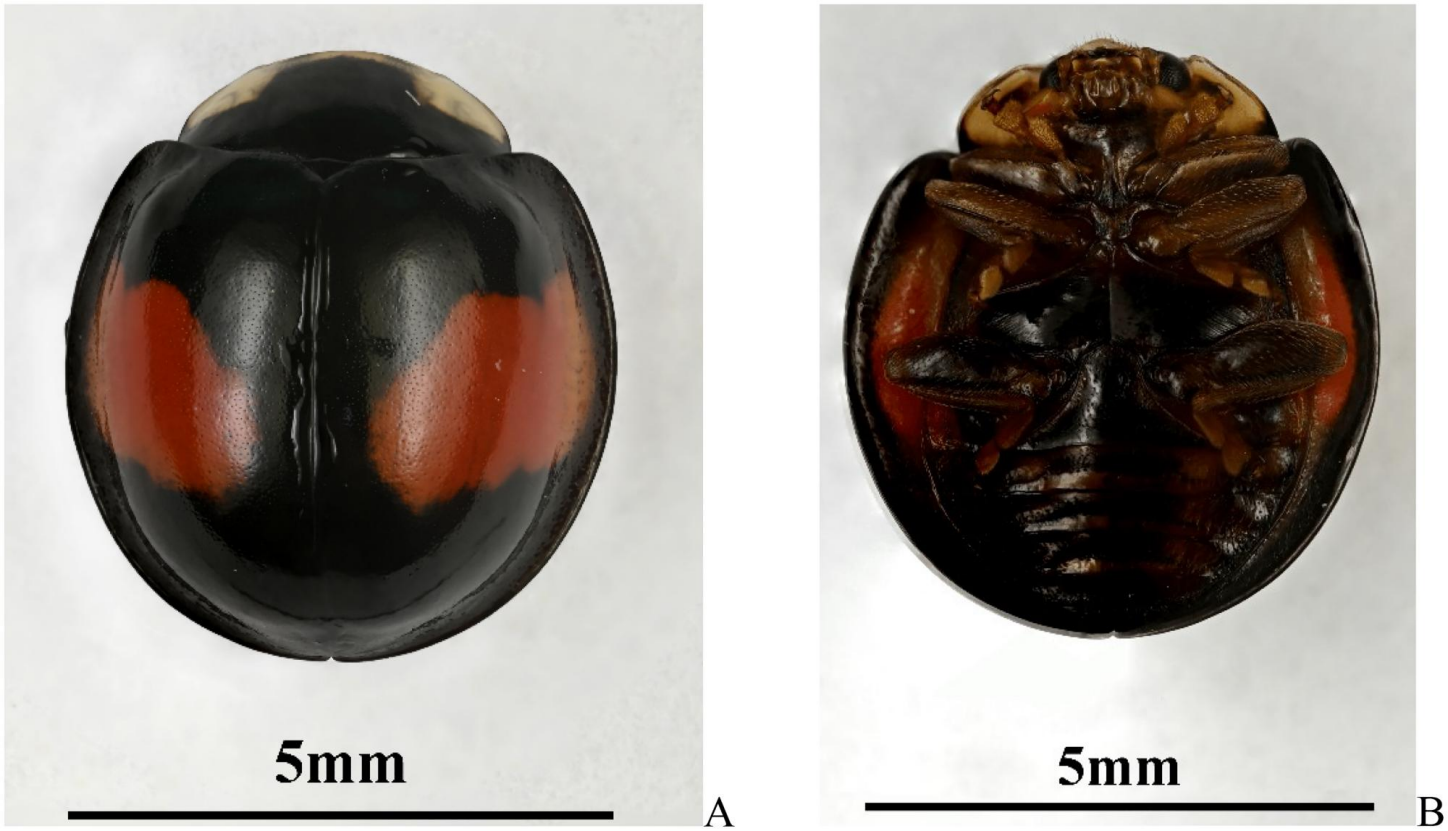
The adult of *C. saucia*. (A) the dorsal view; (B) the ventral view. Photographed by Jing-Qiang zhou.

The *C. saucia* specimens used in this study were collected on September 10, 2024 by Jing-Qiang Zhou at the Broccoli Selenium-enriched Ecological Planting Base, Longgui Township, Shaoguan City, Guangdong Province, China (24°44′N 113°25′E). The specimen (Figure 1) and its genomic DNA are stored at the Insect Collections of Hunan Agricultural University, Changsha, China. The material is cataloged under the voucher code HAUHL143796, with contact information provided to Guo-Hua Huang at ghhuang@hunau.edu.cn for further inquiries or assistance.

## Methods

DNA was extracted from muscle tissues of *C. saucia* adult using the SteadyPure Universal Genomic DNA Extraction Kit (Changsha, China). Subsequent to extraction, clustering of the index-coded samples was performed on a cBot Cluster Generation System using NovaSeq 6000 S4 Reagent Kit (Illumina), and 2 × 150 bp short reads were generated through paired-end sequencing *via* the Illumina NovaSeq 6000 platform. Raw reads underwent filtering using FASTQ (Chen et al. 2018). The complete mitochondrial genome of *C. saucia* was assembled utilizing NOVOPlasty (Dierckxsens et al. 2017) and GetOrganelle v1.7.6 (Jin et al. 2020), with adjustments made post-assembly. The average sequencing depth of the genome was 4782x, with a minimum depth of 2324x and a maximum depth of 10525x (Figure S1). Annotation of the mitochondrial genome was performed through MITOS (Bernt et al. 2013), employing Geneious (version 5.4.2) (Kearse et al. 2012), Final visualization was achieved using CGview, generating a circular map accessible *via* [https://proksee.ca/].

For sequence analysis, mitochondrial genomes from 22 published species within the family Coccinellidae and two outgroup species from the family Endomychidae were retrieved from the NCBI database (Table S1). A total of 13 protein-coding genes (PCGs) were manually extracted and concatenated using Geneious Prime (v2024.0.5). Multiple sequence alignment was performed using MAFFT v1.5.0, with model selection performed through ModelFinder v2.2.0 (Kalyaanamoorthy et al. 2017). The optimal molecular evolution model (GTR + F + I + R4) was chosen for the analysis. A phylogenetic tree was constructed employing IQ-TREE v2.4.0 (Minh et al. 2020), and bootstrap values were calculated with 1000 iterations. The tree topology was visualized using FigTree v1.4.4 and iTOL (https://itol.embl.de/) (Ivica and Peer 2024). Species divergence times were estimated using MCMCTree (Yang and Rannala 2006). Calibration points were selected based on the fossil record of Coccinellidae.

## Results

### Structural organization of the *Coelophora saucia* mitogenome

**Figure 2.**
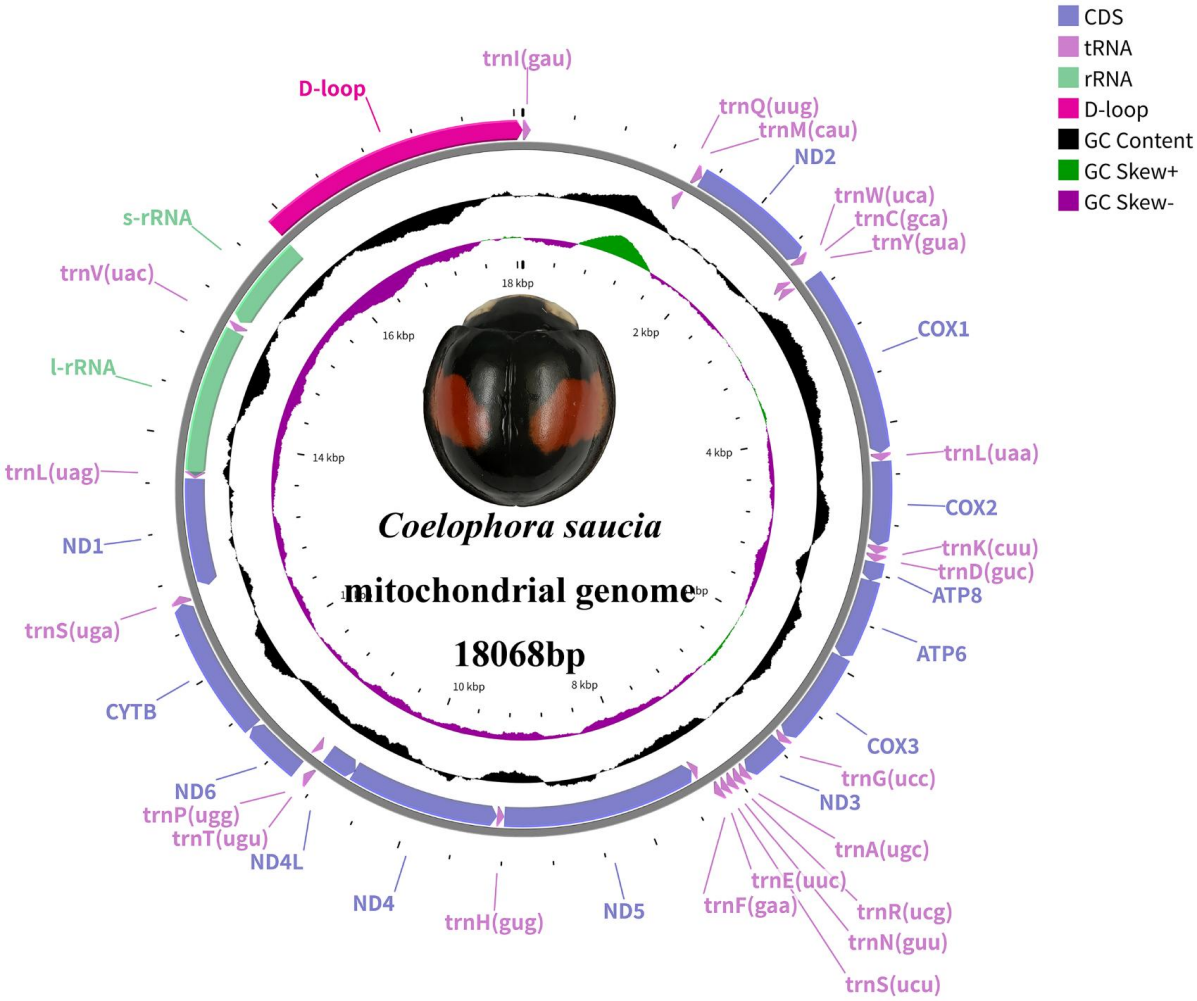
Mitogenome pattern map of coelophora saucia. Genes located within the gray circle are coded into the light strand (L-strand), while those outside the black circle are encoded in the heavy strand (H-strand). The Middle black circle denotes the GC content, while the innermost circle signifies the GC skew.

The complete mitochondrial genome of *C. saucia* (GenBank accession: PQ668616) comprises a circular DNA molecule spanning 18,068 bp. This sequence includes 13 protein-coding genes (PCGs), 22 transfer RNA (tRNA) genes, 2 ribosomal RNA (rRNA) genes, and a single non-coding control region (D-loop) (Table S2). Genomic organization was asymmetric with respect to gene distribution across strands: nine PCGs and fourteen tRNAs were encoded on the heavy strand, while the remaining genes were localized on the light strand (Figure 2).

### Nucleotide composition and codon usage

The overall nucleotide composition exhibited a bias toward purines (*A* = 41.6%, *T* = 37.5%; *C* = 11.9%, *G* = 9.0%), resulting in a GC content of 20.9%. Among the PCGs, twelve utilize ATN (N: A/T/C/G) as initiation codons, while *COX1* uniquely employs AAG. Termination codon analysis revealed TAA/TAG as stop codons for eight PCGs, whereas five PCGs displaying incomplete termination *via* single T residues.

### Non-coding and structural features

The 22 tRNA genes exhibited variable lengths, ranging from 58 bp (*trnR*) to 69 bp (*trnK*). Ribosomal RNA components included a 799 bp 12S rRNA and a 1325 bp 16S rRNA. Intergenic spacers spanned approximately 1–1290 bp between 12 adjacent genes, with one pair of genes exhibiting overlapping regions (1–38 bp), totaling 60 bp of sequence overlap. A non-coding control region (D-loop) measuring 2186 bp was located between the rRNAs and tRNAs.

### Phylogenetic relationships and divergence

**Figure 3.**
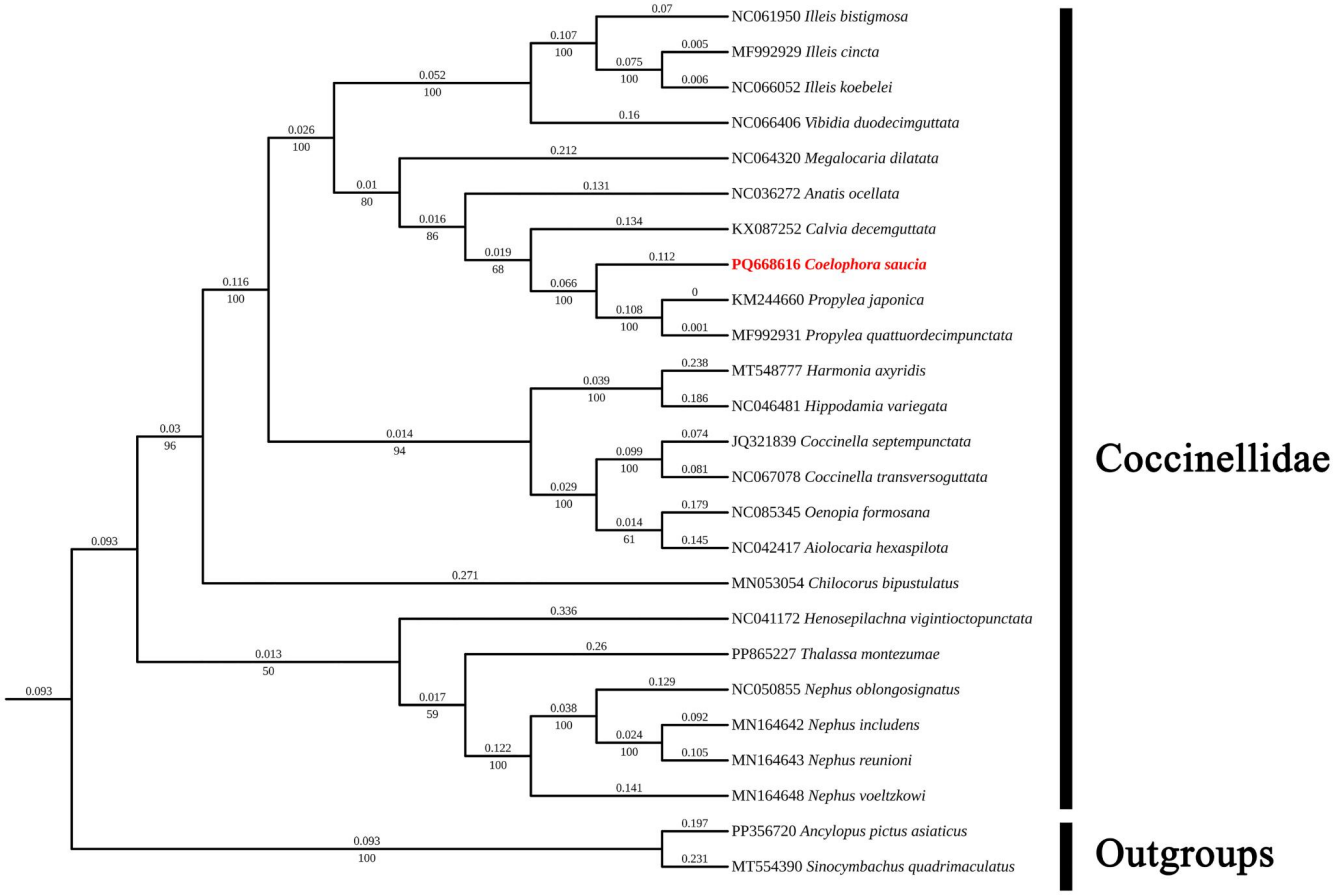
Maximum likelihood (ML) tree of 23 species within the tribe coccinellidae based on 13 PCGs of the mitogenome with 2 endomychidae species as outgroups. The numerical values positioned above the branches denote genetic distances, whereas the values displayed below correspond to bootstrap support percentages.

The molecular phylogenetic tree in Figure 3 was constructed using concatenated sequences of 13 PCGs from *C. saucia*. The results reveal that *C. saucia* is more closely evolutionarily related to the *Propylea* spp. forming a distinct clade with strong phylogenetic support (genetic distance of 0.066, bootstrap value = 100%). These findings are consistent with previous study by Zhou et al. (2019), which further validate the close evolutionary relationships between *C. saucia* and *Propylea* spp. This topology highlights the close phylogenetic affinity between the three species, as evidenced by Bayesian divergence time estimates that indicate a shared most recent common ancestor with the *Propylea* lineage (posterior probability = 1.0).

The high level of genetic similarity between *C. saucia* and *P*. species (average nucleotide identity: 98.2% across PCGs) further corroborates their sister-group relationship. This clade is distinct from other members of the Coccinellidae tribe, with branch lengths suggesting a divergence event approximately 18.01 million years ago, this divergence time estimation is calibrated based fossil records within Coccinellidae, including *Coccinella* spp. (14.12 Ma) *Propylea japonica* (7.15 Ma), *Vibidia duodecimoguttata* (19.20 Ma), and *Illeis koebelei* (1.63 Ma) (Huang et al. 2025). These findings resolve uncertainties in the systematic position of C. *saucia* and extend our understanding of its evolutionary relationships within the broader context of Coccinellidae.

## Discussion and conclusion

The present study reports the first complete mitochondrial genome sequence of *C. saucia*. Unlike previous studies by Zhou et al. (2019), which identified several mitochondrial genes, this study refined the sequence to include a complete mtDNA genome, thereby providing a more accurate representation of the species’ genetic makeup.

The Maximum Likelihood phylogenetic tree robustly placed *C. saucia* as an outgroup to the genus Propylea. This clear topological structure indicates that *C. saucia* is phylogenetically distinct from the core Propylea species, adds nuance to the previous classification proposed by Zhu et al. (2022). Based on comparisons of gene content, AT content, and gene order with other members of the tribe (Coccinellidae), the newly sequenced *C. saucia* mitochondrial genome shows remarkable similarity to *P. quattuordecimpunctata* (MF992931) and *P. japonica* (KM244660). Additionally, it shares several conserved features with other members of the tribe, such as the majority of mitochondrial protein coding genes starting in tRNA regions (Ge et al. 2021). These findings highlight the need for taxonomic review of the tribe, contributing to a better understanding of its evolutionary history. To conclusively resolve the taxonomic status of *C. saucia*, future studies should incorporate a broader sampling of related genera and employ phylogenomic approaches.

## Supplementary Material

Table S1.pdf

Figure S1.jpg

Table S2.pdf

## Data Availability

The genome sequence data that support the findings of this study are openly available in GenBank of NCBI under the accession number PQ668616. The associated BioProject, SRA, and BioSample numbers are PRJNA1187021, SRR31442579, and SAMN44780754, respectively.
