# Supplementary material for: Characterization of the complete mitochondrial genome and phylogenetic analysis of Coelophora saucia (Mulsant, 1850) (Coleoptera: Coccinellidae)
Source: Mitochondrial DNA B Resour. 2025 Nov 10;10(12):1154–8. doi: 10.1080/23802359.2025.2571724 (PMC12604118; doi:10.1080/23802359.2025.2571724)
Supplement: Table S1.pdf [file TMDN_A_2571724_SM4564.pdf]

The Coleoptera mitochondrial genomes used for phylogenetic inference		
**Accessionnumber**	**Species**	**Reference**
NC061950	*Illeis bistigmosa*	Zhu et al. 2023
MF992929	*Illeis cincta*	Song and Li, 2022
NC066052	*Illeis koebelei*	Direct Submission
NC066406	*Vibidia duodecimoguttata*	Direct Submission
NC064320	*Megalocaria dilatate*	Direct Submission
NC036272	*Anatis ocellata*	Direct Submission
KX087252	*Calvia decemguttata*	Direct Submission
PQ668616	*Coelophora saucia*	This study
KM244660	*Propylea japonica*	Tang et al. 2014
MF992931	*Propylea quattuordecimpunctata*	Direct Submission
MT548777	*Harmonia axyridis*	Direct Submission
NC046481	*Hippodamia variegata*	Hao et al. 2019
JQ321839	*Coccinella septempunctata*	Kim et al. 2012
NC067078	*Coccinella transversoguttata*	Direct Submission
NC085345	*Oenopia formosana*	Direct Submission
NC042417	*Aiolocaria hexaspilota*	Seo et al. 2023
MN053054	*Chilocorus bipustulatus*	Song et al. 2020
NC041172	*Henosepilachna vigintioctopunctata*	Direct Submission
PP865227	*Thalassa montezumae*	Iovin et al. 2024
NC050855	*Nephus oblongosignatus*	Magro et al.2020
MN164642	*Nephus includens*	Magro et al.2020a
MN164643	*Nephus reunion*	Magro et al.2020b
MN164648	*Nephus voeltzkowi*	Magro et al.2020c
PP356720	*Ancylopus pictus asiaticus*	Direct Submission
MT554390	*Sinocymbachus quadrimaculatus*	Direct Submission
