# Supplementary figures and images for: Characterization of the complete mitochondrial genome and phylogenetic analysis of Coelophora saucia (Mulsant, 1850) (Coleoptera: Coccinellidae)
Source: Mitochondrial DNA B Resour. 2025 Nov 10;10(12):1154–8. doi: 10.1080/23802359.2025.2571724 (PMC12604118; doi:10.1080/23802359.2025.2571724)

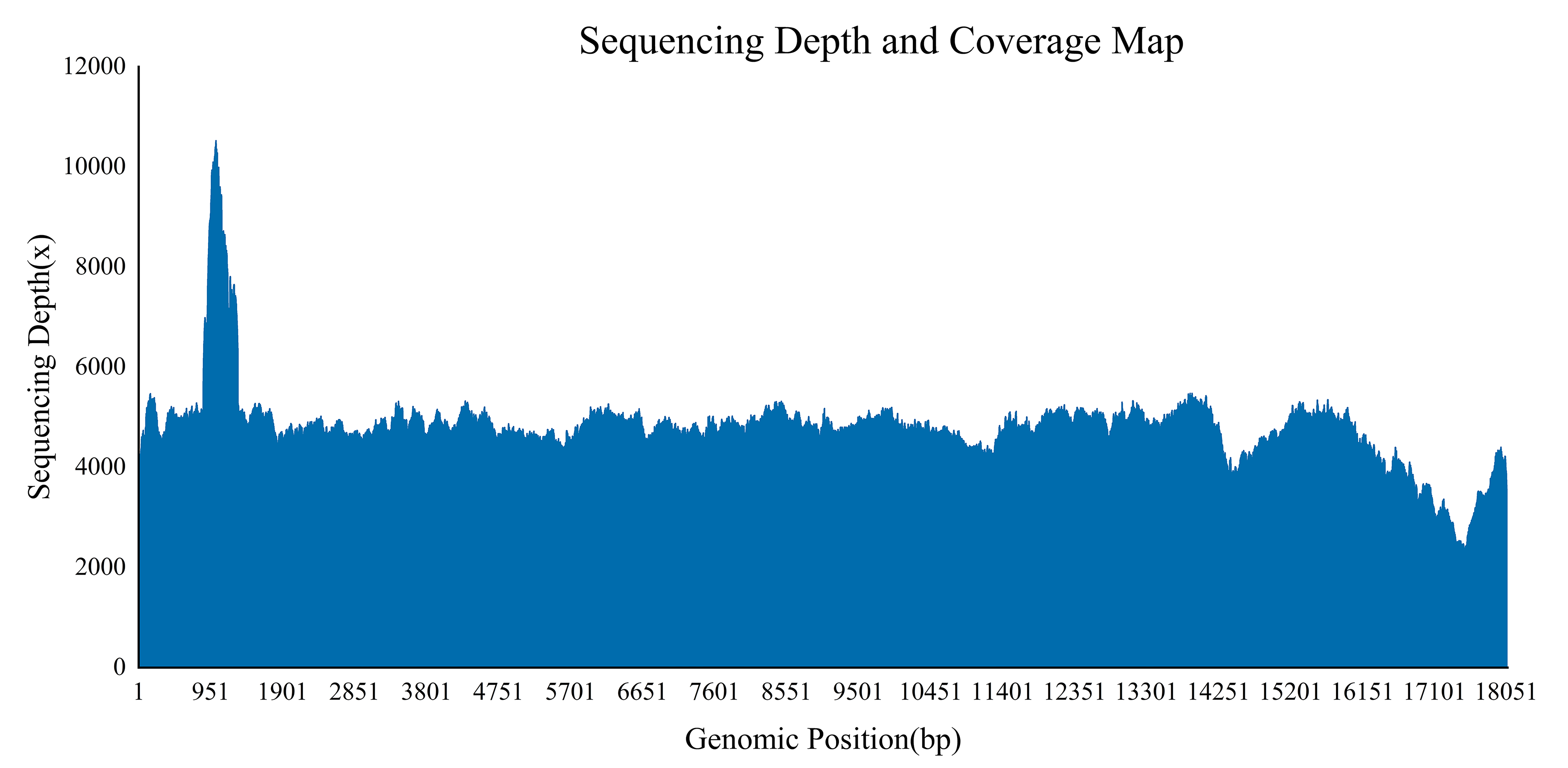

Supplement: Figure S1.jpg [file TMDN_A_2571724_SM4563.jpg]
