# Supplementary material for: Characterization of the complete mitochondrial genome and phylogenetic analysis of Coelophora saucia (Mulsant, 1850) (Coleoptera: Coccinellidae)
Source: Mitochondrial DNA B Resour. 2025 Nov 10;10(12):1154–8. doi: 10.1080/23802359.2025.2571724 (PMC12604118; doi:10.1080/23802359.2025.2571724)
Supplement: Table S2.pdf [file TMDN_A_2571724_SM4562.pdf]

Annotation table of *Coelophora saucia* mitogenome

Gene	Position(bp)	Length(bp)	Direction	Intergenic nucleotides	start/stop codons	AT%
trnI	1-64	64	J	1290		65.6%
trnQ	1355-1423	69	N	9		84.1%
trnM	1433-1500	68	J	0		69.1%
ND2	1501-2508	1008	J	-1	ATT/TAA	80.8%
trnW	2508-2569	62	J	-8		77.4%
trnC	2562-2628	67	N	2		79.1%
trnY	2631-2693	63	N	4		76.2%
COX1	2698-4228	1531	J	0	AAG/T	70.5%
trnL	4229-4292	64	J	0		71.9%
COX2	4293-4971	679	J	0	ATT/T	73.0%
trnK	4972-5040	69	J	0		71.0%
trnD	5041-5104	64	J	0		90.6%
ATP8	5105-5260	156	J	-4	ATT/TAA	87.8%
ATP6	5257-5910	654	J	0	ATA/TAA	77.2%
COX3	5911-6691	781	J	0	ATG/T	73.8%
trnG	6692-6755	64	J	0		87.5%
ND3	6756-7109	354	J	-1	ATA/TAA	79.9%
trnA	7109-7170	62	J	0		82.3%
trnR	7171-7234	64	J	0		79.7%
trnN	7235-7298	64	J	0		79.7%
trnS	7299-7356	58	J	1		84.5%
trnE	7358-7421	64	J	0		92.2%
trnF	7422-7484	63	N	-1		82.5%
ND5	7484-9196	1713	N	0	ATT/TAA	81.1%
trnH	9197-9261	65	N	0		83.1%
ND4	9262-10582	1321	N	-7	ATG/T	81.5%
ND4L	10576-10854	279	N	1	ATG/TAA	83.2%
trnT	10856-10921	66	J	0		86.4%
trnP	10922-10981	60	N	2		75.0%
ND6	10984-11479	496	J	0	ATT/T	84.1%
CYTB	11480-12619	1140	J	1	ATG/TAA	76.1%
trnS	12621-12686	66	J	17		81.8%
ND1	12704-13645	942	N	0	ATT/TAG	79.4%
trnL	13646-13707	62	N	-8		79.0%
16S rRNA	13670-14994	1325	N	1		82.3%
trnV	14996-15059	64	N	24		81.2%
12S rRNA	15084-15882	799	N	0		81.7%
control region D-loop	15883-18068	2186				84.2%
